# Methylome profiling reveals functions and genes which are differentially methylated in serrated compared to conventional colorectal carcinoma

**DOI:** 10.1186/s13148-015-0128-7

**Published:** 2015-09-17

**Authors:** Pablo Conesa-Zamora, José García-Solano, María del Carmen Turpin, Patricia Sebastián-León, Daniel Torres-Moreno, Eduardo Estrada, Anne Tuomisto, Jamie Wilce, Markus J. Mäkinen, Miguel Pérez-Guillermo, Ana Conesa

**Affiliations:** Department of Pathology, Santa Lucía General University Hospital (HGUSL), C/Mezquita s/n, 30202 Cartagena, Spain; Facultad de Ciencias de la Salud, Catholic University of Murcia (UCAM), Murcia, Spain; Francisco de Vitoria University, Madrid, Spain; Department of Bioinformatics and Genomics, Centro de Investigación Príncipe Felipe (CIPF), Yúfera, 3, 46012 Valencia, Spain; Department of Social Psychology and Methodology, Autónoma University, Madrid, Spain; Department of Pathology, University of Oulu, Oulu, Finland; Microbiology and Cell Science, Institute of Food and Agricultural Science, University of Florida, Gainesville, USA

**Keywords:** Serrated carcinoma, Colorectal cancer, Methylome, Microarray analysis, FOXD2, DIO3, CpG island, Pyrosequencing

## Abstract

**Background:**

Serrated adenocarcinoma (SAC) is a recently recognized colorectal cancer (CRC) subtype accounting for 7.5–8.7 % of CRCs. It has been shown that SAC has a worse prognosis and different histological and molecular features compared to conventional carcinoma (CC) but, to date, there is no study analysing its methylome profile.

**Results:**

The methylation status of 450,000 CpG sites using the Infinium Human Methylation 450 BeadChip array was investigated in 103 colorectal specimens, including 39 SACs and 34 matched CCs, from Spanish and Finnish patients. Microarray data showed a higher representation of morphogenesis-, neurogenesis-, cytoskeleton- and vesicle transport-related functions and also significant differential methylation of 15 genes, including the iodothyronine deiodinase *DIO3* and the forkhead family transcription factor *FOXD2* genes which were validated at the CpG, mRNA and protein level using pyrosequencing, methylation-specific PCR, quantitative polymerase chain reaction (qPCR) and immunohistochemistry. A quantification study of the methylation status of CpG sequences in *FOXD2* demonstrated a novel region controlling gene expression. Moreover, differences in these markers were also evident when comparing SAC with CRC showing molecular and histological features of high-level microsatellite instability.

**Conclusions:**

This methylome study demonstrates distinct epigenetic regulation patterns in SAC which are consistent to previous expression profile studies and that *DIO3* and *FOXD2* might be molecular targets for a specific histology-oriented treatment of CRC.

**Electronic supplementary material:**

The online version of this article (doi:10.1186/s13148-015-0128-7) contains supplementary material, which is available to authorized users.

## Background

Serrated adenocarcinoma (SAC) has been accepted in the latest WHO classification of tumours of the digestive system as a new subtype of colorectal cancer (CRC) [1]. Criteria for its histologic diagnosis have been proposed [2] and recently validated in a series of 81 cases [3]. Its frequency ranges from just 7.5 to 8.7 % of all CRCs [2, 3], but according to 2012 Globocan statistics and given the high incidence of CRC from all cancers (9.7 %), SAC would have an incidence similar to that of multiple myeloma (0.8 %) (http://globocan.iarc.fr). It has been shown that SAC has distinct histological and molecular features and a worse prognosis than conventional carcinoma (CC) [3]. Accordingly, our group observed that SAC, compared to CC, displays a higher frequency of adverse histological features at the invasive front [4] that differs in the expression pattern of adhesion molecules [5] and oncogene mutation [6]. Additionally, two previous studies on mRNA profiling have revealed that SAC showed a higher representation of morphogenesis-, hypoxia-, cytoskeleton- and vesicle-transport-related functions and also an over-expression of HIF-1α, fascin1 (actin-bundling protein associated with invasion) and the antiapoptotic gene hippocalcin and a downregulation of the morphogenesis-related proteins EPHB2 and PTCH [7, 8]. Moreover, SAC differs from those CRCs displaying histological features of high-level microsatellite instability (hMSI-H) in terms of oncogene mutation prevalence, MSI status and MLH1 expression [6, 8].

A two-arm model has been proposed to explain the progression of the serrated pathway, both of which may progress to SAC. Cancers developing from this pathway may show high- or low-level microsatellite instability (MSI-H and MSI-L, respectively) or may be microsatellite stable (MSS) [9]. Sporadic CRC characterized by MSI-H and BRAF mutation displays typical histological features compared to SAC [10–13] and is considered a different endpoint of the serrated pathway [14].

In the serrated pathway, inhibition of apoptosis and subsequent inactivation of DNA repair genes by promoter methylation appear to play an important role [1, 2]. Although aberrant cytosine-phospho-guanine (CpG) methylation has been proposed as the typical leading mechanism for serrated carcinogenesis [14], there is no study analysing methylome profiling in SAC.

In this study, we have analysed the methylome profile of SAC with the aim of:

- Evaluating the differentially methylated biological functions of SAC and comparing them with previous studies including mRNA profiling [7, 8].
- Validating the genes differentially methylated in SAC in comparison with CC at the CpG, mRNA and protein level.
- Studying the presence of the identified biomarkers in hMSI-H and comparing them with those found in SAC.

## Results

As a prerequisite for a matched group, CC did not show significant differences in terms of demographic and clinico-pathologic features with SAC in the training and validation sets included in the study (Table 1).

**Table 1:** Demographic and pathological features of the study cases

	Training set			DNA validation set (MSP and pyroseq)			RNA validation set (qPCR)			Protein validation set (IHC)		
	SAC	CC		SAC	CC		SAC	CC		SAC	CC	
	*n* = 39 (%)	*n* = 34 (%)	*p* value	*n* = 59 (%)	*n* = 44 (%)	*p* value	*n* = 18 (%)	*n* = 25 (%)	*p* value	*n* = 42 (%)	*n* = 49 (%)	*p* value
Gender												
Female	20 (51.3)	16 (47.1)		29 (49.2)	21 (47.7)		8 (44.4)	9 (36.0)		30 (71.4)	28 (57.1)	
Male	19 (48.7)	18 (52.9)	0.719	30 (50.8)	23 (52.3)	0.846	10 (55.6)	16 (64.0)	0.5763	12 (28.6)	21 (42.9)	0.157
Age [SD]	71.6 [10.0]	72.3 [7.6]		72.0 [9.5]	70.3 [8.4]	0.348	69.4 [9.8]	72.6 [11.0]	0.331	69.9 [6.8]	70 [10.7]	0.959
Location												
Proximal	26 (66.7)	21 (61.8)		34 (57.6)	26 (59.1)		10 (55.6)	14 (56.0)		23 (54.8)	26 (53.1)	
Distal /rectum	13 (33.3)	13 (44.1)	0.554	25 (42.3)	18 (40.1)	0.882	8 (44.4)	11 (44.0)	0.977	19 (45.2)	23 (46.9)	0.871
Dukes’ stage												
A	4 (10.3)	4 (11.8)		6 (10.2)	4 (9.1)		2 (11.1)	3 (12.0)		7 (16.7)	8 (16.3)	
B	13 (33.3)	15 (44.1)		24 (40.7)	18 (40.9)		4 (22.2)	13 (52.0)		15 (35.7)	17 (34.7)	
C	17 (43.6)	15 (44.1)	0.875	29 (49.2)	22 (50.0)	0.983	12 (66.7)	9 (36.0)	0.094	20 (47.6)	24 (49.0)	0.992
WHO grade												
High	5 (12.8)	2 (5.9)		3 (5.1)	1 (2.3)		0	0		0	2 (4.1)	
Low	34 (87.2)	32 (94.1)	0.315	56 (94.9)	43 (97.3)	0.465	18 (100)	25 (100)	NA	42 (100)	47 (95.9)	0.186
Type												
Non-mucinous	34 (87.1)	30 (88.2)		51 (84.4)	39 (88.6)		16 (88.9)	22 (88.0)		36 (58.7)	41 (83.7)	
Mucinous	5 (12.8)	4 (11.8)	0.891	8 (13.6)	5 (11.4)	0.740	2 (11.1)	3 (12.0)	0,929	6 (14.3)	8 (16.3)	0.788

*SAC* serrated adenocarcinoma, *CC* conventional carcinoma, *MSP* methylation-specific PCR, *CpG pyroseq* CpG island pyrosequecing, *qPCR* quantitative polymerase chain reaction, *IHC* immunohistochemistry, *SD* standard deviation, *WHO* World Health Organisation

### Differentially methylated functions

**Fig. 1:**
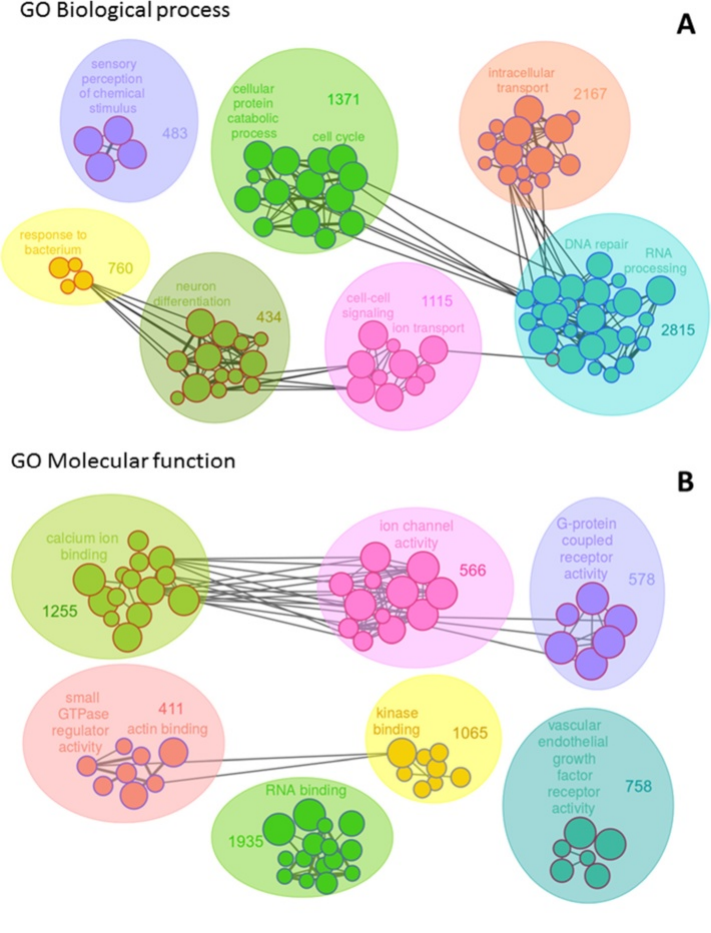
GO plots representing significant Gene Ontology biological process **a** and molecular functions **b** differentially methylated in SAC compared to CC. Each node shows a significant function and its size the grade of significance. *Red contours* around the nodes indicate that this function is more represented in SAC whereas *blue signifies* CC. Nodes are grouped in clusters showing similar functions. The number for each cluster shows the amount of unique genes for this cluster. The different functions were grouped according to the concordance Kappa value based on the number of shared genes between functions (only *lines* representing a Kappa > 0.2 are depicted and *line thickness* indicates higher Kappa)

Noisy methylation measurements and the high number of genes tested in the array, which imposes strong *p* value corrections, usually hamper the detection of significant methylation differences at the gene level. As this may be the case in our study, we opted for an alternative, yet well-established approach to identify regulated functions in high “omics” experiments, namely the Gene Set Enrichment Analysis approach (GSEA) which relies on the ordering of genes according to a molecular phenotype, as differential expression or methylation, rather than on a gene selection based on a pre-defined *p* value cutoff. We used the GSEA-related method Fatiscan [8] to analyse our data. This approach revealed a considerable number of GO terms differentially methylated in SAC vs. CC: 86 GO biological processes (BP), 31 GO CC and 69 GO molecular functions (MF). Differentially methylated activities were related to ion binding, intracellular transport, actin binding, GTPases and kinase signaling, neural markers, DNA repair and VEGF signaling amongst others. Figure 1 shows the FatiScan annotated function corresponding to the GO biological process and molecular functions, and Additional file 1 represents box plots indicating the number and the percentage of genes belonging to each of these functions. Additional file 2 shows the GO plots and box plots corresponding to the GO cellular component category. In contrast, only 7 GO biological processes, 8 cellular components and 11 molecular functions were differentially methylated when comparing Spanish SAC and Finnish SAC tumour cases (data not shown).

### Differentially methylated genes

The analysis of the methylome microarray data identified 15 differentially methylated genes, 14 of which were more methylated in SAC than in CC (Table 2). No significant methylated genes were observed when comparing normal SAC and CC mucosa or Spanish and Finnish serrated cases.

**Table 2:** Differentially methylated genes between serrated adenocarcinoma and conventional carcinoma obtained from the methylome analysis

Symbol	Gene name and protein function	> meth in:	Fold change	Raw *p* value	Adj. *p* value
DIO3	Type III iodothyronine deiodinase. Inactivation of thyroid hormone	SAC	209.939.101.331.552	7.41E+05	0.0001
FOXD2	Forkhead box protein D2. Transcription factor	SAC	0.889154808110723	7.14E+07	0.0066
OR4N5	Olfactory receptor, family 4, subfamily N, member 5. G protein-coupled receptor	SAC	135.181.723.252.813	8.99E+08	0.0452
RIOK3	RIO kinase 3. Cytoskeleton rearrangement	SAC	0.635422579240145	9.83E+08	0.0452
WASF3/WAVE3	Wiskott-Aldrich syndrome protein family, member 3. Cytoskeleton rearrangement	SAC	120.522.262.330.085	1.38E+09	0.0467
OR51G1	Olfactory receptor, family 51, subfamily G, member 1. G protein-coupled receptor	SAC	168.046.340.201.344	1.81E+09	0.0467
BPIFA2/SPLUNC2	Short palate, lung and nasal epithelium carcinoma associated 2. Lipopolysaccharide binding	SAC	0.911123350424225	2.24E+09	0.0467
ATP6V1C1	ATPase, H+ transporting, lysosomal 42 kDa, V1 subunit C1. Intracellular protein sorting and endocytosis	SAC	160.360.515.634.696	2.29E+09	0.0467
NIPAL4	NIPA-like domain containing 4. Membrane receptor	SAC	153.320.826.961.118	2.67E+09	0.0467
LRRK2	Leucine-rich repeat kinase 2. GTPase and kinase	SAC	170.637.559.235.708	2.72E+09	0.0467
QPRT	Quinolinate phosphoribosyltransferase. Catabolism of endogenous excitotoxin to neurons	CC	−0.698324044955379	3.28E+09	0.0467
PRR5	Proline rich 5 (renal). mTOR pathway	SAC	0.892188635181469	3.63E+09	0.0467
XKR4	Kell blood group complex subunit-related family, member 4. Apoptosis	SAC	0.737099384900948	3.66E+09	0.0467
FAM19A5	Family with sequence similarity 19 (chemokine (C-C motif)-like), A5. Regulation of immune and nervous cells	SAC	0.734137349204086	3.69E+09	0.0467
POT1	protection of telomeres 1. Telomere maintenance	SAC	0.551207360675112	3.81E+08	0.0467

*SAC* serrated adenocarcinoma, *CC* conventional carcinoma, *> meth in* CRC subtype showing higher methylation for that gene

The differentially methylated genes that we found encode transcription factors (FOXD2), kinases (RIOK3), G protein-coupled receptor and GTPases (OR4N5, OR51G1, LRRK2) which are involved in hormone regulation (DIO3), cytoskeleton and vesicle transport (RIOK3, WASF3, ATP6V1C1), apoptosis (XKR4), morphogenesis (FOXD2), regulation of nervous cells (FAM19AS, QPRT) and telomere maintenance (POT1) amongst others (Table 2).

### Validation of methylated sites by MSP and pyrosequencing

Based on the extent of the differential methylation grade, the importance of the biological functions, the design of suitable primers and the availability of antibodies, we decided to validate *DIO3* and *FOXD2* by methylation-specific PCR (MSP) and pyrosequencing, respectively, and by quantitative polymerase chain reaction (qPCR) and immunohistochemistry (IHC) for both. As regards the first two, the distribution of CpG sites and the flanking sequence of the *DIO3* and *FOXD2* CpG islands make the validation by MSP more feasible in the case of DIO3 (CpG sites more condensed) and by pyrosequencing in the case of FOXD2 (CpGs more scattered).

Consistent with the microarray results, MSP revealed that DIO3 CpGs were more methylated in SAC than in CC cases (36/59, 61.1 vs. 14/44, 31.8 %; *p* = 0.003), Additional file 3 showing representative results. When the correlation of *DIO3* methylation was evaluated with oncogene mutation and MSI status, no significant associations were found (data not shown) except for a tendency of BRAF mutation with *DIO3* methylation (BRAF mutated, 10/13 (79.9 %) vs. BRAF WT, 24/51 (47.1 %); *p* = 0.054).

**Fig. 2:**
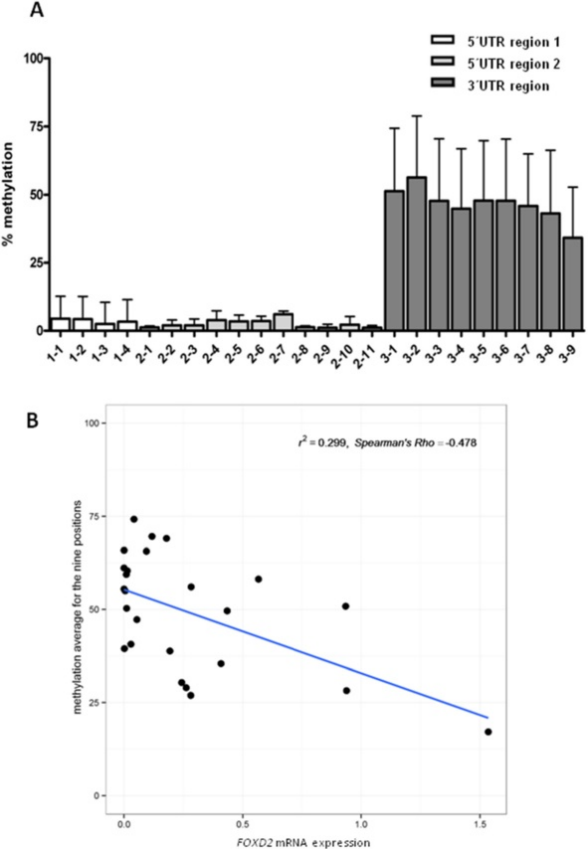
Mean percentage of the different CpG sites found in the first (4 sites) and second (11 sites) 5′ UTR and in the 3′ UTR regions of FOXD2 (**a**). Correlation graphic between the methylation percentage of FOXD2 3′ UTR CpG sites and FOXD2 mRNA expression by qPCR (**b**)

Concerning FOXD2, CpG sequence analysis revealed three different clusters of CpG sites, two in the 5′ UTR and one in the 3′ UTR. From these three regions, only the 3′ UTR, which comprised nine CpG sites, was found to be substantially methylated (Fig. 2a). The mean and SD of the methylation percentage for each CpG site and study group is shown in Additional file 1. Interestingly, normal tumour-adjacent mucosa showed a lower percentage of methylation (mean = 31.19) than the tumoural group (mean = 52.81), this difference being statistically significant (*F* (1.106) = 28.636, *p* < 0.001, partial = 0.213). The non-tumoural cases had a more similar methylation pattern than the tumoural cases (Levene’s *F* > 16, *p* < 0.001) (Additional file 4A).

**Fig. 3:**
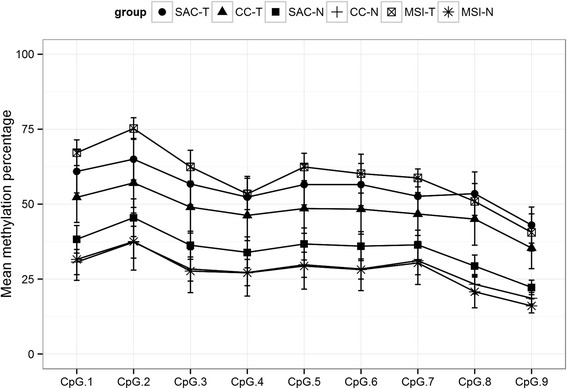
Mean methylation percentages of the nine CpG sites of the 3′UTR region of *FOXD2* in the different study groups

Figure 3 and Additional file 3 show that SAC displays a higher percentage of methylation compared to that observed for CC in each of the nine CpG sites, although not reaching statistical significance. In order to test which variable was associated with higher *FOXD2* methylation, a bivariate analysis was performed. Higher *FOXD2* methylation was associated with tumoural specimens, KRAS, BRAF and exon 20 PIK3CA mutation and MSI-H status (Table 3). Therefore, specimen status, *KRAS*, *BRAF* and MSI were used as predictors for the multivariate analysis whereas exon 20 PIK3CA mutation was excluded as the sample size was less than 15. Additional file 5 indicates that only tumoural status and MSI remained significant independent predictors. For the normal and MSS cases, the model predicts a 32.8 % occurrence of methylation in R3 with an increase of 17.1 points for tumoural cases and 13.7 for MSI cases. Moreover, external validation with the TCGA database was also successful showing that MSI-H expressed less *FOXD2* than MSI-L/MSS colon carcinomas (*p* = 0.001) (Additional file 6).

**Table 3:** Differences in percentage of methylation in FOXD2 3′ UTR for the two groups defined with each binary variable

Variable	*n*1	*n*2	Mean1	Mean2	Eequal variances	Difference (1–2)	gl	*t*	*p* values
*Source*	31 normal	90 tumoural	32.35	52.69	No	−20.35	112	*−7.094*	*<0.001*
Nationality	98 Spanish	23 Finnish	47.62	46.89	No	0.72	29	.128	0.899
Sex	32 female	59 male	47.98	46.96	Yes	1.02	119	.262	0.794
M^a^	106 0 s	12 1 s	47.38	50.69	Yes	−3.31	116	−.517	0.606
*KRAS*	85 native	34 mutated	44.89	53.74	Yes	−8.85	117	*−2.095*	*0.038*
*BRAF*	100 native	18 mutated	44.93	62.11	No	−17.18	48	*−5.247*	*<0.001*
PI3K exon10^a^	90 native	5 mutated	46.91	58.55	Yes	−11.65	93	−1.254	0.213
PI3K exon20^a^	92 native	3 mutated	46.91	66.37	No	−19.47	3	−3.692	*0.038*
*MSI*	102 MSS	16 MSI	44.65	63.58	No	−18.93	70	*−6.887*	*<0.001*

^a^When the sample size is <15 in any of the groups, *t* test should not be interpreted. The Mann-Whitney’s *U* test was used for the variables *M*, *PI3K exon10* and *PI3K exon20*. No significant differences were found for any of them

### Validation by qPCR

**Fig. 4:**
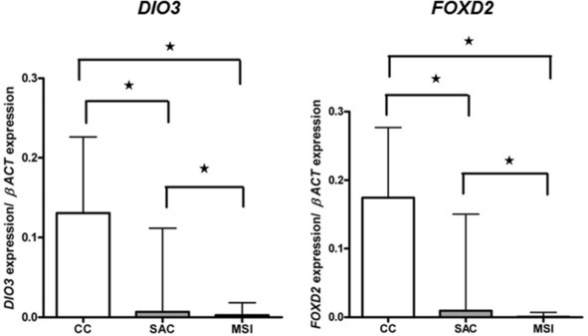
qPCR results of the mRNA expression of *DIO3* and *FOXD2* genes in SAC, CC, and hMSI-H tumoural tissue. *Asterisk* indicates statistical significance

With the aim of finding out whether the *DIO3* and *FOXD2* hypermethylation affected gene activation, an analysis of the mRNA expression by quantitative PCR was performed. As shown in Table 4, all the compared medians were significantly different. The tumoural and non-tumoural groups differed in the expression of *DIO3* and *FOXD2*, the non-tumoural group showing a lower median for both variables (0.154 ± 0.18 vs. 0.054 ± 0.134; *p* = 0.043 and 0.281 ± 0.473 vs. 0.095 ± 0.301; *p* = 0.013, respectively, Additional file 4B). Amongst tumoural cases, SAC displayed lower expression medians than CC for both genes (*DIO3*, 0.007 ± 0.095 vs. 0.131 ± 0.155; *p* = 0.02 and *FOXD2*, 0.01 ± 0.253 vs. 0.174 ± 0.331; *p* = 0.041) (Fig. 4). Additionally, a positive relationship between *DIO3* and *FOXD2* expression was observed. For the whole group (tumoural and non-tumoural, serrated and conventional), Pearson’s correlation coefficient was *r* = 0.792 (*p* < 0.001, *n* = 52); this value indicating that the expression of both genes shares almost 63 % of their variance (*R*^2^ = 0.627). If we consider only the tumoural cases, it was *r* = 0.751 (*p* < 0.001, *n* = 43), both genes sharing 56 % of their variance (*R*^2^ = 0.564) (Additional file 6). In fact, CRC data deposited in the TCGA data sets confirmed this direct correlation as significant (*p* = 0.037) [15] as retrieved via www.cbioportal.org.

**Table 4:** *DIO3* and *FOXD2* mRNA expression assessed by quantitative PCR

		Tumoural cases			All cases	
		SAC	CC	hMSI-H	Non-tumoural	Tumoural
	*n*	18	25	8	9	43
	*Median* ± *SD*	0.007 ± 0.095	0.131 ± 0.155	0.0021424 ± 0.008	0.154 ± 0.18	0.054 ± 0.138
DIO3	Mann-Whit. *U*		131	51^a^/29^b^		110
	*p*		0.020	0.243^a^/0.002^b^		0.043
	*Median* ± *SD*	0.01 ± 0.253	0.174 ± 0.331	0.0007 ± 0.003	0.281 ± 0.473	0.095 ± 0.301
FOXD2	Mann-Whit. *U*		142	33^a^/7^b^		91
	*p*		0.041	0.031^a^/<0.001^b^		0.013

*SAC* Serrated adenocarcinoma, *CC* Conventional carcinoma, *hMSI-H* colorectal carcinoma showing typical molecular and histological features of MSI-H

^a^SAC vs. hMSI-H comparison; ^b^CC vs. hMSI-H comparison

In order to test if 3′ UTR *FOXD2* CpG sites might be regulating *FOXD2* expression, we performed a Spearman’s correlation analysis which showed that all these sites revealed negative *r* values indicating an inverse relationship between methylation and mRNA expression and statistically significant correlations except for CpG2 (*p* < 0.01) and CpG9 sites (*p* = 0.084). This finding was also observed when representing the mean of the methylation of the nine CpG positions with the qPCR results (Fig. 2b, Additional file 7).

### Validation by immunohistochemistry

**Fig. 5:**
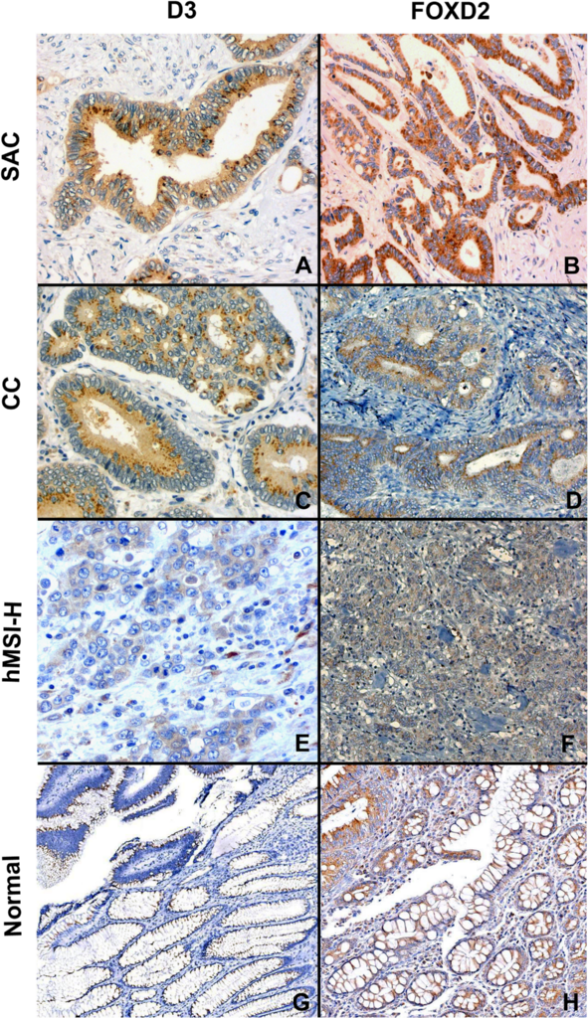
Immunohistochemical expression of D3 and FOXD2 in SAC (**a**, **b**), CC (**c**, **d**), hMSI-H (**e**, **f**), and normal mucosa including an adenoma area in the upper left corner (**g**, **h**). ×20 magnification and H-E counterstained

In order to investigate whether differential methylated status of *DIO3* and *FOXD2* in SAC compared to CC could have an effect on protein expression within the tissue cells, immunohistochemistry for both D3 and FOXD2 was performed. Both markers showed a granular cytoplasmic staining which was more evident at the luminal border (Fig. 5). Whereas no significant differences were observed in the staining distribution of D3 when SAC vs. CC were compared, FOXD2 staining was more diffuse in SAC (*p* = 0.013) (Table 5). In addition, SAC showed higher D3 and FOXD2 intensity staining than CC (*p* = 0.051, *p* = 0.054), being statistically significant when strong intensity was compared to combined weak and moderate staining (D3, 45.2 vs. 22.4 %; *p* = 0.018, FOXD2, 54.8 vs. 30.6 %; *p* = 0.020).

**Table 5:** Immunohistochemical expression of D3 and FOXD2 proteins in SAC, CC and hMSI-H CRC

			SAC (*n* = 42)		CC (*n* = 49)			hMSI-H (*n* = 13)		
Protein		n	n	%	n	%	*p* value	n	%	*p* value†
D3	distribution	A	7	16,7	8	16,3		0	0,0	
		B	8	19,0	16	32,7	0.144^*^	1	7,7	0.049^*^
		C	27	64,3	25	51,0	0.313	12	92,3	0.132
	intensity	1	11	26,2	22	44,9		7	53,8	
		2	12	28,6	16	32,7	0.018^*^	5	38,5	0.013^*^
		3	19	45,2	11	22,4	0.051	1	7,7	0.039
FOXD2	distribution	A	0	0,0	6	12,2		7	53,8	
		B	6	14,3	13	26,5	0.008^*^	3	23,1	<0.0001^*^
		C	36	85,7	30	61,2	0.013	3	23,1	<0.0001
	intensity	1	2	4,8	2	4,1		11	84,6	
		2	17	40,5	32	65,3	0.02^*^	2	15,4	0.0005^*^
		3	23	54,8	15	30,6	0.054	0	0,0	<0.0001

*SAC* Serrated adenocarcinoma, *CC* Conventional carcinoma, *hMSI-H* colorectal carcinoma showing typical molecular and histological features of MSI-H

^*^Fisher p value calculated when grouping the first two categories, i.e. A + B or 1 + 2

†SAC vs. hMSI-H comparison

### Biomarker comparison between SAC and hMSI-H

As shown in Fig. 3, hMSI-H tumour cases were more methylated in *FOXD2* than SAC and CC which was not unexpected since the MSI-H feature was associated with, and was an independent factor for, *FOXD2* methylation (Table 3, Additional file 5). Consequently, qPCR analysis revealed that *FOXD2* expression was less in hMSI-H than that in SAC (*p* = 0.030) or CC (*p* < 0.001) and that of *DIO3* less in hMSI-H than that in CC (*p* = 0.002) but without reaching statistical significance in the comparison with SAC (*p* = 0.243) (Table 4). Interestingly, immunohistochemical analysis revealed that hMSI-H showed less D3 and FOXD2 expression than SAC and CC, both in staining distribution and intensity, especially for FOXD2 (Table 5; Fig. 4)

## Discussion

Altered methylation patterns not only serve as important diagnostic and prognostic markers but are also aetiological factors in colonic carcinogenesis [16]. Whereas the pathogenic sequence leading to conventional carcinoma has been extensively studied, the molecular events responsible for the serrated pathway are poorly understood. SAC is considered as one endpoint of the serrated pathway and, since its recognition by the WHO as a different CRC entity, several studies have aimed to characterize it [2–8]. O’ Brien et al. investigated the methylation status of five genes (hMLH1, MGMT, MINT1, MINT2, p16) comprising the so-called CpG-island methylation phenotype (CIMP) in serrated carcinomas and precursor polyps [17] and found that higher CIMP levels (four or more markers positive) were significantly more frequent in advanced stages of the serrated pathway. However, no previous studies have analysed the SAC methylome by means of high-throughput techniques.

Our study suggests that SAC shows differential methylated functions and genes compared to CC and, moreover, the 186 GO differentially methylated functions found are consistent with previously reported differentially expressed functions (GTPases, cytoskeleton, morphogenesis, apoptosis, vesicle transport and neuronal regulation functions) obtained from two microarray studies comparing the mRNA profile of 5 [7] and 11 [8] SACs with that of CCs. In addition, these functions are also supported by different studies characterizing the histological, immunohistochemical and molecular features of SAC [3–6, 12]. Noteworthy, Spanish and Finnish SAC cases only showed 26 differentially methylated GO functions and no differentially methylated gene, thus supporting the criteria for its diagnosis [11] and for a common pathogenic mechanism despite the differences in environmental and genetic background.

As regards the differentially methylated genes, it is important to emphasize that none were observed in the comparison of non-tumoural SAC vs. matched non-tumoural CC, therefore supporting the absence of a distinct and relevant baseline methylation alteration. The comparison between SAC and CC showed 15 genes differentially methylated, 14 of which were more methylated in SAC, thus highlighting the involvement of aberrant methylation in SAC compared to CC. In fact, SAC, in comparison to CC, was more frequently CIMP high than CC (11/37, 30 % vs. 3/32, 9 %; *p* = 0.0035) (unpublished observations). The differentially methylated genes found are consistent with the functions previously assigned to SAC in microarrays studies [7, 8] like GTPase activity (*OR51G1* and *LRRK2*), cytoskeleton (*RIOK3* and *WASF3*), vesicle transport (*ATP6V1C1*), mTOR pathway (*PRR5*), apoptosis (*XKR4*), membrane-associated functions (*NIPAL4* and *DIO3*), morphogenesis (*DIO3* and *FOXD2*), immune response (*FAM19A5* and *WASF3*) and neural markers (*QPRT*).

The only gene more methylated in CC than in SAC was *QPRT* which codes for an enzyme that was found to detoxify quinolinate, a potent endogenous neuron toxin that is elevated in the brain of patients with neurodegenerative disorders such as Alzheimer's disease [18]. In this respect, previous studies from our group reported the differential expression of neural regulation processes and the upregulation of specific genes such as hippocalcin, a sensor protein that participates in preventing the calcium-driven apoptosis in neurons, of SAC compared to CC [8]. Therefore, this finding suggests that SAC tumoral cells use neuroprotective mechanisms to prevent apoptosis, a common hallmark of this tumour which is responsible for its saw-shaped growth pattern. The validation of *DIO3* and *FOXD2* at the DNA, mRNA and protein level was carried out based on its level of significance, CpG site distribution and reagent availability. The MSP study confirmed that *DIO3* was more methylated in SAC than in CC, and qPCR confirmed that this might result in a decrease in the mRNA level of expression. DIO3 protein, also known as D3, is the type 3 deiodinase which inactivates the tissue effects of thyroid hormones, these playing a pivotal role in the regulation of cell proliferation and morphogenesis of the intestine as observed in animal models [19]. In fact, D3 is elevated in various colon cancer cell lines as well as in human colorectal cancer tissues compared to their normal counterparts and its expression is a result of the canonical Wnt/β-catenin pathway activation [20]. Therefore, the less-methylated status and the increased expression of *DIO3* in CC cases suggest an involvement of D3 in the downstream transcriptional effects of β-catenin which characterize the CC, but not the SAC carcinogenic process. As opposed to CC, nuclear β-catenin is not expressed in the tumour invasive front [6], nor it also observed in a serrated mice model [21], thus suggesting a lack of implication of this protein in the epithelial-mesenchymal transition in SAC. Other reports have demonstrated that D3, under the control of the Sonic Hedgehog (Shh) pathway, promotes cell proliferation in basal cell carcinomas [22]. Interestingly, whereas the increase in *DIO3* methylation in SAC was confirmed with decreasing mRNA expression, the IHC study revealed a higher intensity cytoplasmic staining of D3 in this type of tumour compared to CC. D3 is an integral membrane protein most of which is extracellular thus allowing ready access to circulating thyroid hormones. It has been reported that the way it is inactivated is by internalization from plasma membrane to early endosomes [23]. The granular cytoplasmic staining observed in our immunohistochemical experiments could suggest a negative feedback mechanism of methylation-driven silencing of *DIO3* gene when D3 accumulates in the cytoplasm or alternative mRNA transcripts which may not be regulated by this CpG methylation. Possible impairment of D3 trafficking to the membrane or accelerated internalization in SAC could be responsible for this observation. In fact, vesicle transport and cytoskeleton are amongst the differentially expressed functions obtained from the comparison between SAC and CC.

Much less is known about FOXD2, a member of the family of the well-conserved winged helix forkhead transcriptional factors. Previous reports in animal models have demonstrated a role for it in morphogenesis, possibly through Shh signaling [24], and in regulating sensitivity to cAMP in T lymphocytes [25]. Noteworthy, the only two studies of FOXD2 in humans are related to cancer; in one, a locus including the *FOXD2* was found to be deleted in meningioma [26] and, in the other, the protein was found to be more highly expressed in prostate cancer and lymph node metastases compared to normal prostate [27]. In our study, we validated the microarray result by qPCR showing that FOXD2 was less expressed in SAC than in CC. By quantifying the relative methylation of FOXD2 CpG island, there was a consistent trend although not reaching statistical significance. Nevertheless, we observed that a region of the 3′ UTR, but not 5′ UTR, appears to be involved in the control of FOXD2 expression and, in fact, we have shown that methylation in this region is strongly correlated with decreased mRNA expression. Moreover, this FOXD2 methylation is related to microsatellite instability, thus the reason why the methylation results did not reach significance as there were MSS CRCs amongst SAC cases. Differences on *FOXD2* RNA expression are more profound and consistent than those observed in its methylation, thus suggesting that additional factors to methylation such gene deletion could be influencing *FOXD2* expression. Interestingly, *FOXD2* maps to chromosome 1p32-34, a locus frequently deleted in the serrated pathway [28]. Whether FOXD2 could be acting as a tumour suppressor gene in MSI-H CRC deserves future studies. FOXD2 showed an increased cytoplasmic expression in SAC compared to CC and, despite being a transcriptional factor, it was not detected in the nucleus of any cases. Hence, as with D3, a negative feedback of cytoplasmic FOXD2 on gene silencing may be implicated. It is important to stress that protein expression assessed by immunohistochemistry does not give information on protein functionality or isoform type. The increased staining of D3 and FOXD2 could be a result of the translation of alternative gene transcripts with a different regulation and stability. In this context, the positive correlation observed between *DIO3* and *FOXD2* expression and prior evidence of the participation of these two proteins in the Shh pathway might justify a common behaviour in protein expression and functionality. In fact, according to TCGA database, *DIO3* expression is associated with only 12 genes, FOXD2 being one of them [15].

Our results also show that SAC and hMSI-H are different CRC entities, the latter showing a higher *FOXD2* methylation percentage and lower *FOXD2* mRNA expression. The immunohistochemical study showed that hMSI-H, as opposed to SAC, displayed a significant lack of staining for both D3 and FOXD2, thus suggesting a direct effect of methylation upon protein expression and highlighting further molecular differences between these two histological CRC subtypes considered as two endpoints of the serrated pathway.

## Conclusions

- SAC and CC are different CRC in terms of methylated functions, thus reinforcing the concept that SAC is characterized by distinct defects in the regulation of morphogenesis, apoptosis, neural markers and Wnt/β-catenin pathway activation.
- *DIO3* and *FOXD2* are two novel genes that are differentially methylated and expressed in CC, SAC and another endpoint of the serrated polyps pathway as it is the CRC showing molecular and histology features of MSI-H, and thus, they may serve as novel interesting diagnostic markers, pathogenic routes tracers or molecular targets.
- This study also reports for the first time the association of *FOXD2* methylation with CRC development and shows which CpGs seem to be involved in gene expression regulation.

## Methods

### Patients and tumour samples

The clinico-pathological features of the patients have been previously reported [3, 4]. Approval for the study was granted by the Santa Lucia University Hospital Ethical Board, and informed consent was obtained from the patients. SACs were diagnosed on the basis of prior established criteria (epithelial serrations, clear or eosinophilic cytoplasm, abundant cytoplasm, vesicular nuclei, absence of, or less than 10 % necrosis of the total surface area, mucin production and cell balls and papillary rods in mucinous areas of a tumour [2]) and so for CCs [13]. Frozen samples of 39 SACs were retrieved from the Santa Lucia University Hospital, Cartagena Spain (*n* = 21) and the Oulu University, Finland (*n* = 18) for CpG methylation profiling and, in addition, adjacent normal mucosa was analysed from 16 SACs (12 Spanish and four Finnish cases). A control group of 34 CCs frozen samples matched with SACs for gender, age, location, Dukes’ stage, WHO grade and mucinous pattern was selected from the same tumour banks (25 Spanish and nine Finnish cases) accompanied by mucosal tissue in 14 of these (10 Spanish and 4 Finnish). MSI-H was present in 20 % and 2.9 % of SAC and CC cases, respectively. The mutation rate in KRAS and BRAF oncogenes, which was previously assessed [6], was 48.5 and 3 % for CC, 33.3 and 33.3 % for SAC and 11.1 and 66.7 % for hMSI-H, respectively. Validation by qPCR was performed on the methylome series and on an additional set of 24 SACs and 12 CCs frozen tumour cases. Paraffin blocks of 42 SAC and 49 CC cases, included in previous studies [6, 8], were used for immunohistochemical validation of the microarray results. Clinico-pathological features of the cases are shown in Table 1. Additionally, a previously described series of 13 CRCs [6, 8] showing MSI-H molecular and histological features (mucinous, signet-ring cell, and medullary carcinoma, tumour infiltrating and peritumoural lymphocytes, “Crohn-like” inflammatory response, poor differentiation, tumour heterogeneity and “pushing” tumour border [10]) termed hMSI-H were also studied. These hMSI-H were found mostly in females (69.2 %), mean age 70.5 (±10.0), located in proximal colon, showing Dukes’ B (38.5 %) and C (61.5 %) stage, morphological WHO low grade and 15.4 % displaying a mucinous pattern in more than 50 % of the tumour. MSI status was previously determined, according to the manufacturer’s instructions, in all CRC cases using the MSI Analysis System, version 1.2 provided by Promega (Madison, USA) which includes fluorescence-labeled primers for co-amplification of seven markers including five mononucleotide repeat markers (BAT-25, BAT-26, NR-21, NR-24 and MONO-27) and two pentanucleotide ones (Penta C and Penta D) [6].

### DNA extraction

A volume of approximately 10 mm^3^ was extracted from each frozen tissue using the disposable sterile biopsy punch. DNA was extracted following the manufacturer’s instructions (QIAGEN, Hilden, Germany). Briefly, tissue was disrupted and homogenized in ATL buffer using a Tissueruptor (QIAGEN) incubated with proteinase K, and the homogenate was subjected to automatic DNA extraction using the Qiacube equipment and the QiaAmp DNA Mini Kit (cat n°:51306), both provided by QIAGEN.

### Bisulfite treatment and DNA methylation assay

HumanMethylation450K BeadChip (Illumina, Inc., San Diego, CA), using Infinium HD Methylation assay for genome-wide DNA methylation screening, was employed. In brief, genomic DNA (1000 ng) from each sample was bisulfite converted with the EZ DNA Methylation Kit (Zymo Research, Orange, CA) according to the manufacturer’s recommendations. Bisulfite-treated DNA was isothermally amplified at 37 °C (20–24 h), and the DNA product was fragmented by an endpoint enzymatic process, then precipitated, resuspended, applied to an Infinium Human Methylation450K BeadChip (Illumina, San Diego, CA, USA) and hybridized at 48 °C (16–24 h). The fluorescently stained chip was imaged by the Illumina i-SCAN and Illumina’s Genome Studio program (Methylation Module) was used to analyse BeadArray data to assign site-specific DNA methylation *β* values to each CpG site.

### Preprocessing of Methylation data

Processing of raw data was done using R packages. Probes with a low detection *p* value (*p* < 0.01) in more than 95 % of the samples and those measuring SNPs or mapping in X or Y chromosomes were removed and normalization followed a three-step procedure. Firstly, a colour bias adjustment was applied using the methylumi R-package [29]. Then, wateRmelon [30] R-package was used to perform between-sample normalization by equalization of type I and type II backgrounds followed by separated quantile normalization of methylated and unmethylated intensities. Finally, A BMIQ [31] intra-sample normalization procedure, included in the wateRmelon R-package, was applied to correct the bias of type II probe values.

### Functional profiling

Functional profiling of the differentially methylated genes was performed using the FatiScan method included in the Babelomics [32, 33] web suite. The Gene Ontology (GO) [34] database was used in this functional profiling analysis.

Results of functional profiling were represented by two graphs for each considered GO category: biological process, molecular function and cellular component. Significant GO terms of each category are clustered applying hclust function included on R [35], using Cohen’s kappa value [36] as a measure of the similarity between two terms. This value measures the agreement between two GO terms as regards shared and exclusive items between them.

### Differential methylation analysis

The analysis of differentially methylated genes was performed using limma [32] R-package. Data were fitted to a linear model and differential methylated genes were identified by using the empirical Bayes method included in the package. If the comparison was done between paired samples, a moderated paired *t* test was applied. A FDR-corrected *p* value of 0.05 was used as the threshold to select differentially methylated genes.

### MSP

Converted DNA from the study cases was subjected to MSP CpG islands analysis. Information on primer sequences and annealing temperatures is provided as Additional file 3. After amplification, PCR products were subjected to electrophoresis using the QIAxcel equipment and the QIAxcel DNA High Resolution Cartridge (ref: 929002, QIAGEN, Hilden, Germany).

### Pyrosequencing

DNA methylation signatures of three different CpG island regions were analysed and quantified using pyrosequencing. Details of primer sequences and PCR are provided in Additional file 3. PCR products were verified using the QIAxcel DNA high-resolution electrophoresis system. Pyrosequencing of methylated sites was performed using the PyroMark Q24 (QIAGEN) according to the manufacturer’s protocol. The methylation level was assessed using the PyroMark Q24 2.0.6 Software (QIAGEN) by which the methylation percentage (mC/mC + C) for each CpG was calculated. The results are presented as the percentage (mean ± SD) of the different CpG sites studied for each of the three regions analysed whose sequences and relative positions are shown as Additional file 7.

### Quantitative PCR for assessing mRNA expression

RNAs from 18 SACs and 25 CCs, including those from the training set, were extracted with the miRNeasy kit (ref:217004, QIAGEN) and used for validation by qPCR. The retrotranscriptase reaction was performed from a total of 1 μg of DNAseI-treated RNA using the DyNAmo cDNA synthesis Kit (ref:F470L) provided by Thermo Scientific (Rockford, IL), and information on the qPCR experiment is provided as Additional file 3. The relative quantitation was done by the 2-ΔCt method using β-actin as the housekeeping gene.

### Immunohistochemistry

The validation subset consisted of 63 SC and 64 CC cases matched for gender, age and location, and a representative area of each tumour was selected by one of us (JGS) for a tissue microarray (TMA) construction as previously described [5]. Details on the immunohistochemistry procedure are provided as Additional file 3.

TMA sections of 2.5 μm were stained with anti-D3 and anti-FOXD2 antibodies after confirming a homogeneous tumour cell staining in whole tissue sections.

These markers were evaluated by considering a staining intensity (1 = none or weak staining, 2 = moderate, 3 = strong) and a staining area score (A < one third, B = between one and two thirds, C > two thirds) in a given area. For statistical analysis, both intensity and distribution were considered.

### Statistical analysis of validation data

For the analysis of quantification of methylated DNA sequences, the data correspond to a split-plot design with one between-subject factor defining six independent groups of cases (SAC, CC, hMSI-H; tumoural and non-tumoural) and one within-subject factor (CpG sites) defining nine repeated measures for every case. Accordingly, we performed two ANOVA SPF-p-q. The first compared the means of the tumoural vs. non-tumoural groups in each of the nine different CpG sites, and the second the means of the six different groups in these sites. For checking the relationship between methylation percentage and binary variables, the *t* test for independent samples and the Mann-Whitney’s *U* test were used. Statistical significance in the immunohistochemistry study was assessed using Pearson *χ*^2^ or Fisher´s exact test when indicated. Descriptive statistics were computed for real-time PCR. External validation of *FOXD2* expression and *DIO3-FOXD2* correlation was performed using the TCGA database for colon carcinomas (*n* = 324) [15]. Statistical analysis was performed using the SPSS (Version 22, Chicago, IL) package.

## Availability of supporting data

The data set supporting the results of this article are available in the GEO repository, GSE68060 in http://www.ncbi.nlm.nih.gov/geo/info/linking.html.

## Additional files

Additional file 1:
**Box plots representing the GO biological process (A) and molecular functions (B) differentially methylated between SAC and CC.** Each colour indicates functions belonging to the same cluster, bar length the number of genes mapping this function and the percentage their contribution to the total of genes included in the function. Additional file 2:
**Significant Gene Ontology cellular components differentially methylated between SAC and CC.** In the GOplot (A), each node shows a significant function and its size the grade of significance. Red contours around the nodes indicate that this function is more represented in SAC whereas blue signify CC. Nodes are grouped in clusters showing similar functions. The number for each cluster shows the amount of unique genes for this cluster. The different functions are grouped according to the concordance Kappa value based on the number of shared genes between functions (only lines representing a Kappa > 0.2 are depicted and line thickness indicates higher Kappa). In the box plot (B), each colour indicates the functions belonging to the same cluster. Bar length represents the number of genes mapping this function, and the percentage indicates the contribution of these genes to the total of genes included in the GO function. Additional file 3:
**Primer sequences used and PCR conditions for the validation by MSP (including a representative result), CpG pyrosequencing (including the result table), quantitative PCR and details on immunohistochemistry and statistical analysis.**
Additional file 4:
**Comparison of non-tumoural compared to tumoural cases in terms of methylation percentage of the nine CpGs at the 3′ UTR** ***FOXD2*** **region (A) and mRNA** ***DIO3*** **and** ***FOXD2*** **expression (B).** Note the different standard deviation from these two groups in A. Asterisk indicates statistical significance. Additional file 5:
**Multivariate analysis of the clinicopathological and molecular factors associated with FOXD2 methylation (A);** ***DIO3*** **and** ***FOXD2*** **mRNA expression in hMSI-H tumoural and normal specimens (B) and external validation using TCGA database showing that MSI-H expressed less FOXD2 than MSI-L/MSS colon carcinomas (C).**
Additional file 6:
**Positive correlation between mRNA expression of DIO3 and FOXD2 in both non-tumoural and tumoural tissues.**
Additional file 7:
**Distribution of the CpG sites studied in the** ***FOXD2*** **gene and correlation table of the methylation percentages at the 3´ UTR CpG sites with** ***FOXD2*** **mRNA expression.**

## Abbreviations

CC: conventional carcinoma
CpG: cytosine-phospho-guanine
CRC: colorectal carcinoma
hMSI-H: colorectal carcinoma showing typical molecular and histological features of MSI-H
IHC: immunohistochemistry
MSI-H: high-grade microsatellite instability
MSP: methylation-specific PCR
MSS: microsatellite stability
qPCR: quantitative polymerase chain reaction
SAC: serrated adenocarcinoma
SD: standard deviation
WHO: World Health Organisation
