# Supplementary figures and images for: Methylome profiling reveals functions and genes which are differentially methylated in serrated compared to conventional colorectal carcinoma
Source: Clin Epigenetics. 2015 Sep 17;7(1):101. doi: 10.1186/s13148-015-0128-7 (PMC4574063; doi:10.1186/s13148-015-0128-7)

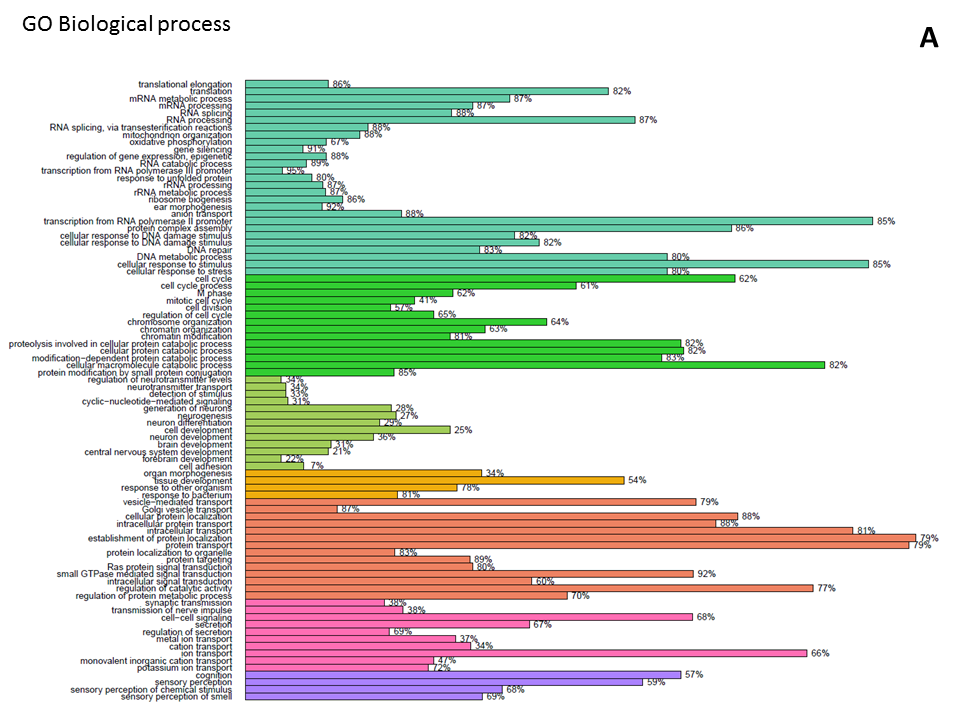

Supplement: Additional file 1: — Box plots representing the GO biological process (A) and molecular functions (B) differentially methylated between SAC and CC. Each colour indicates functions belonging to the same cluster, bar length the number of genes mapping this function and the percentage their contribution to the total of genes included in the function. [file 13148_2015_128_MOESM1_ESM.zip › Additional file 1A.TIF]

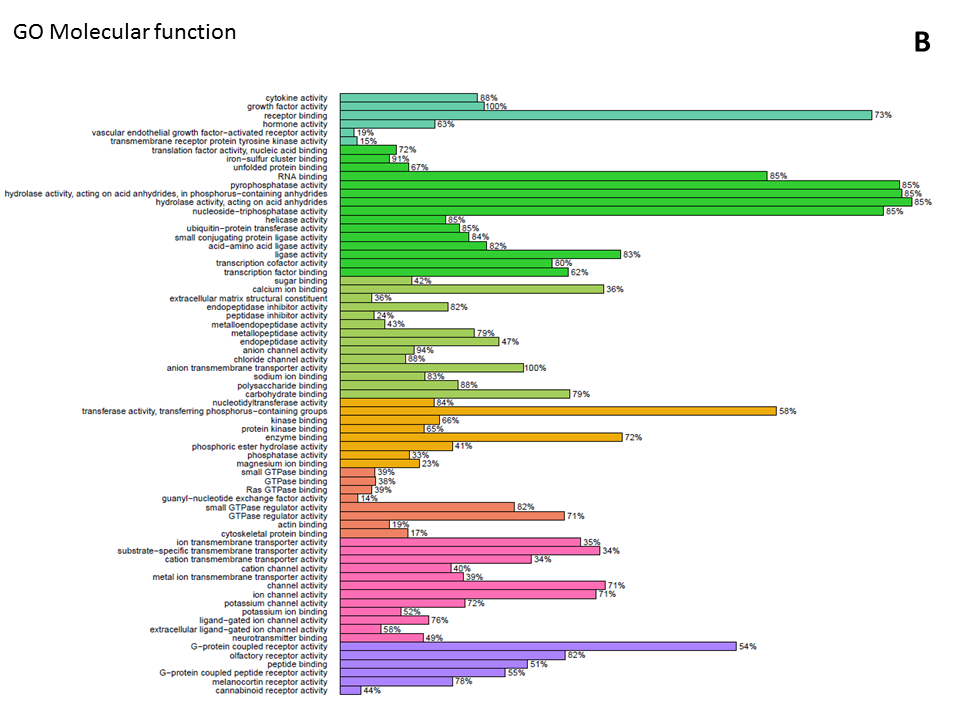

Supplement: Additional file 1: — Box plots representing the GO biological process (A) and molecular functions (B) differentially methylated between SAC and CC. Each colour indicates functions belonging to the same cluster, bar length the number of genes mapping this function and the percentage their contribution to the total of genes included in the function. [file 13148_2015_128_MOESM1_ESM.zip › Addtional file 1B.TIF]

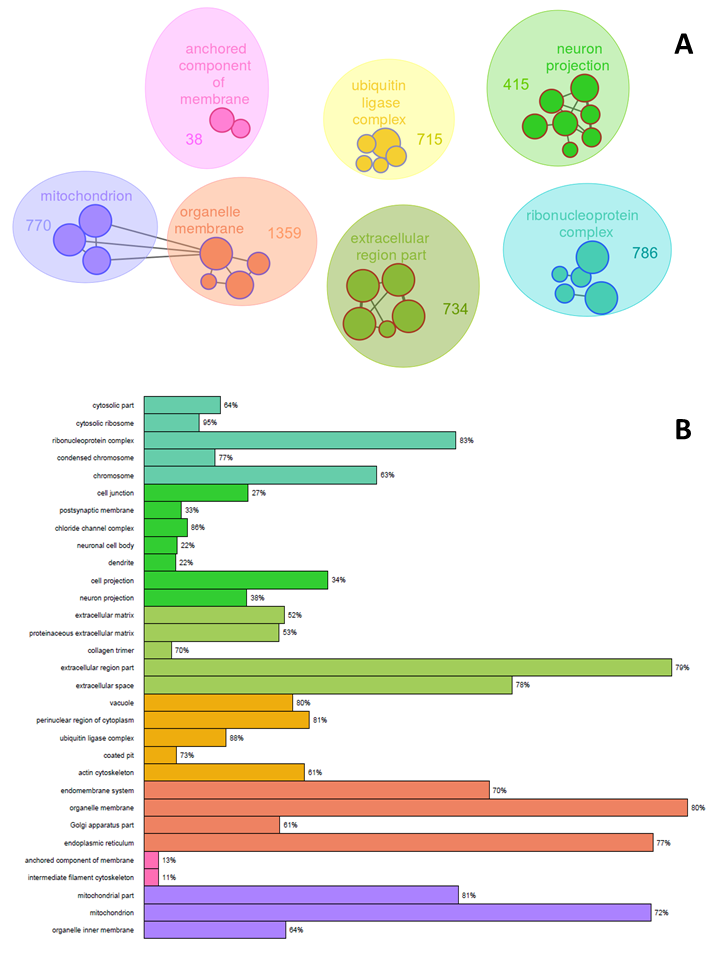

Supplement: Additional file 2: — Significant Gene Ontology cellular components differentially methylated between SAC and CC. In the GOplot (A), each node shows a significant function and its size the grade of significance. Red contours around the nodes indicate that this function is more represented in SAC whereas blue signify CC. Nodes are grouped in clusters showing similar functions. The number for each cluster shows the amount of unique genes for this cluster. The different functions are grouped according to the concordance Kappa value based on the number of shared genes between functions (only lines representing a Kappa > 0.2 are depicted and line thickness indicates higher Kappa). In the box plot (B), each colour indicates the functions belonging to the same cluster. Bar length represents the number of genes mapping this function, and the percentage indicates the contribution of these genes to the total of genes included in the GO function. [file 13148_2015_128_MOESM2_ESM.tif]

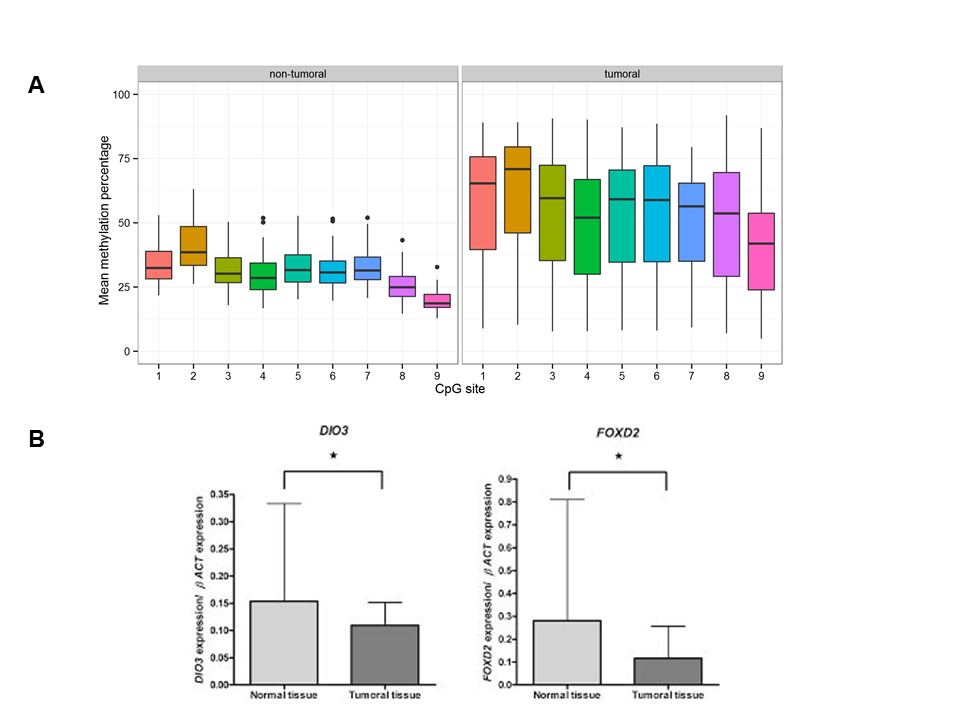

Supplement: Additional file 4: — Comparison of non-tumoural compared to tumoural cases in terms of methylation percentage of the nine CpGs at the 3′ UTR FOXD2 region (A) and mRNA DIO3 and FOXD2 expression (B). Note the different standard deviation from these two groups in A. Asterisk indicates statistical significance. [file 13148_2015_128_MOESM4_ESM.tif]

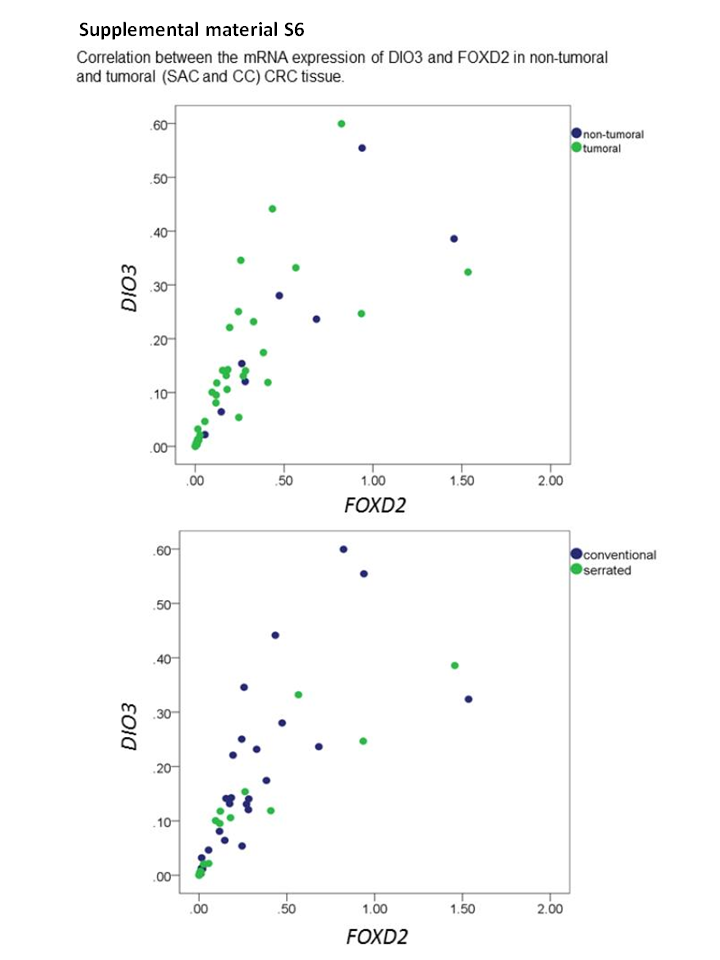

Supplement: Additional file 6: — Positive correlation between mRNA expression of DIO3 and FOXD2 in both non-tumoural and tumoural tissues. [file 13148_2015_128_MOESM6_ESM.tif]
