# Supplementary material for: Methylome profiling reveals functions and genes which are differentially methylated in serrated compared to conventional colorectal carcinoma
Source: Clin Epigenetics. 2015 Sep 17;7(1):101. doi: 10.1186/s13148-015-0128-7 (PMC4574063; doi:10.1186/s13148-015-0128-7)
Supplement: Additional file 3: — Primer sequences used and PCR conditions for the validation by MSP (including a representative result), CpG pyrosequencing (including the result table), quantitative PCR and details on immunohistochemistry and statistical analysis. [file 13148_2015_128_MOESM3_ESM.doc]

**Supplemental material S3**

Primer sequences, PCR conditions and immunohistochemistry procedure used for the different molecular techniques carried out.

**1.** Methylation specific PCR (MSP) primers for selective amplification of methylated (M) and unmethylated (U) sequence of the *DIO3* gene. Primers of the promoter region analysed were drawn up using MethPrimer [1]

| Primer name | Primer sequence 5´-3´ | Annealing  temperature | Amplicon size (bp) |
| --- | --- | --- | --- |
| DIO3-MF | TTCGGGTAGTTTAGTTTTTTAGACG | 60ºC | 256 |
| DIO3-MR | ATACGAAACAACTCCCTAACTCGTA |
| DIO3-UF | TGGGTAGTTTAGTTTTTTAGATGG | 58ºC | 254 |
| DIO3-UR | ATACAAAACAACTCCCTAACTCATA |

**1.1.** Representative results of methylation-specific PCR assay showing amplification of unmethylated (U) and methylated (M) sequences. Lanes 1-8 correspond to CC and 9-12 to SAC tumoral cases


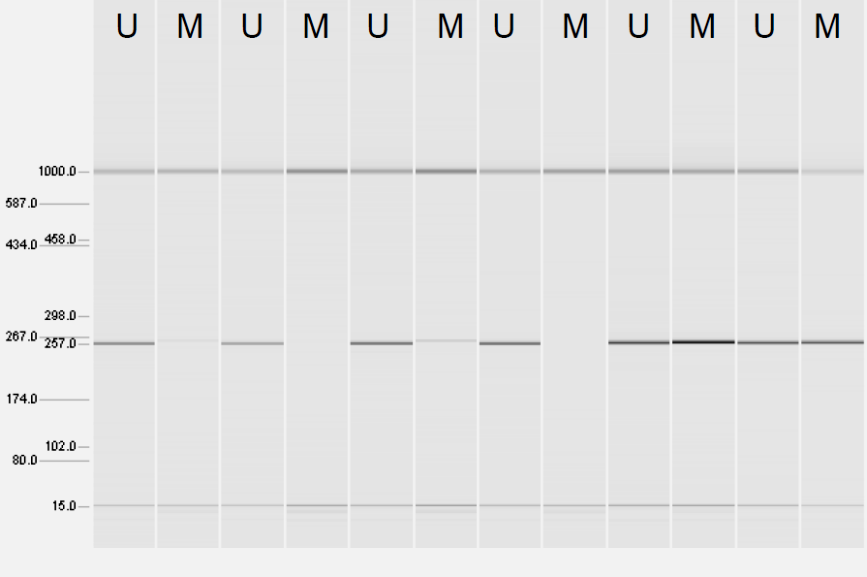


Control alignment markers were included at 1,000 and 15 bp.

**2.** Pyrosequencing for the relative quantitation of CpG methylation in 5´and 3´untranslated regions (UTRs) of *FOXD2*

Bisulfite-converted DNA was previously amplified by PCR using Hot-Start GoTaq polymerase (Promega, Madison, WI) under the following conditions: 1ul of DNA, 4ul of 5X polymerase buffer, 0.2mM dNTPs, 0.6 mM MgCl2, 0.3μM of either biotin-labelled forward or reverse primers and 0.05U/μl Hot-start Go Taq Flexi polymerase (Promega). PCR protocol was performed as follows: initial denaturation at 94ºC for 2min, 35 cycles of 94ºC 10s, 53 or 59ºC 10s and 72ºC 50s and a final extension step of 72ºC 7min.

| Region | Primer name | Primer sequence 5´-3´ | Annealing  Temperature | Amplicon size (bp) |
| --- | --- | --- | --- | --- |
| 1 (5´UTR) | FOXD2-R1F | GGGAAGTTATTTAATGAGGGTTATATAGT | 53ºC | 176 |
|  | FOXD2-R1R | [Btn]CAAATTAAACCCATTTCAACCTCTAAAACA |  |  |
|  | FOXD2-R1seq | GGAGGGTAGTTGGGAT | | |
|  | Sequence to analyse | TTYGGTTTYGTAYGTTYGAGTTTTAGTTATTGTG | | |
| 2  (5´UTR) | FOXD2-R2F | AGGGAGTATAGGGTGTAGGA | 59ºC | 260 |
|  | FOXD2-R2R | [Btn]AACCTCAACCCCTAATCC |  |  |
|  | FOXD2-R2seq | GTATAGGGTGTAGGAG | | |
|  | Sequence to analyse | YGGYGGYGAAGATAAGGGTTYGTTTTYGGTTATTYGAGTTTAGTTTTYGTYGYGGY  GGYGGTTTGTTTT | | |
| 3  (3´UTR) | FOXD2-R3F | [Btn]TAGGGAGTTTATTTATGAAGTTTTTAGA | 53ºC | 190 |
|  | FOXD2-R3R | CCCCCTACTTTTATTTCTCAAACTA |  |  |
|  | FOXD2-R3seq | CTCAAACTATAAAAAATCTTAACC | | |
|  | Sequence to analyse | ACRACTTCTTCRCRTAACCCCRAACCTATCRCCCTAAACRATAAAACCCRCTTAAT  CCCAACTATCTACRAAATTCTATACRTAAAACCTAAAATACC | | |

[Btn]: Biotin labelled

T and A: Converted unmethylated citosines after bisulfite treatment of direct and reverse sequences, respectively.

Y: Ambiguity code for C/T

R: Ambiguity code for G/A (complementary to C/T)

**2.1.** Methylation percentages in nine CpG sites of the 3´UTR region of *FOXD2* gene.

| Group |  | CpG1 | CpG2 | CpG3 | CpG4 | CpG5 | CpG6 | CpG7 | CpG8 | CpG9 |
| --- | --- | --- | --- | --- | --- | --- | --- | --- | --- | --- |
| SAC-T (*n*=38) | *Mean* | 62.3 | 66.4 | 58.0 | 53.5 | 57.8 | 57.8 | 53.7 | 53.5 | 43.0 |
|  | *SD* | 21.4 | 20.4 | 20.8 | 21.1 | 20.4 | 21.3 | 17.6 | 22.8 | 18.9 |
| CC-T (*n*=34) | *Mean* | 52.9 | 57.7 | 49.6 | 46.7 | 49.1 | 48.8 | 47.2 | 45.6 | 35.4 |
|  | *SD* | 25.4 | 24.9 | 26.3 | 25.7 | 25.1 | 25.4 | 21.8 | 26.5 | 20.5 |
| SAC-N (*n*=14) | *Mean* | 38.3 | 45.5 | 36.3 | 33.9 | 36.7 | 36.0 | 36.4 | 29.3 | 22.2 |
|  | *SD* | 8.8 | 12.0 | 8.9 | 9.9 | 10.2 | 9.2 | 9.4 | 7.0 | 4.6 |
| CC-N (*n*=12) | *Mean* | 30.7 | 37.3 | 28.3 | 27.2 | 29.8 | 28.4 | 31.1 | 23.3 | 18.6 |
|  | *SD* | 7.4 | 9.4 | 7.1 | 7.7 | 7.4 | 5.9 | 8.3 | 5.7 | 3.4 |
| MSI-T (*n*=5) | *Mean* | 67.2 | 75.2 | 62.4 | 53.4 | 62.4 | 60.2 | 58.7 | 50.9 | 40.6 |
|  | *SD* | 4.9 | 4.1 | 6.3 | 6.0 | 5.2 | 7.4 | 3.4 | 6.8 | 7.1 |
| MSI-N (*n*=5) | *Mean* | 31.6 | 37.5 | 27.7 | 27.1 | 29.3 | 28.2 | 30.4 | 20.8 | 16.1 |
|  | *SD* | 8.0 | 10.9 | 8.2 | 8.8 | 8.8 | 8.0 | 8.2 | 6.1 | 2.7 |
| Non-tumoral (*n*=31) | *Mean* | 34.2 | 41.0 | 31.8 | 30.2 | 32.8 | 31.8 | 33.4 | 25.6 | 19.8 |
| SAC-N+CC-N+MSI-N | *SD* | 8.7 | 11.3 | 8.9 | 9.3 | 9.4 | 8.6 | 8.9 | 7.1 | 4.5 |
| Tumoral (*n*=77) | *Mean* | 58.4 | 63.1 | 54.6 | 50.5 | 54.2 | 54.0 | 51.2 | 49.8 | 39.5 |
| SAC-T+CC-T+MSI-T | *SD* | 23.1 | 22.4 | 23.1 | 22.7 | 22.4 | 22.9 | 19.3 | 24.0 | 19.3 |
| Total (*n*=108) | *Mean* | 51.5 | 56.8 | 48.0 | 44.6 | 48.1 | 47.6 | 46.1 | 42.9 | 33.8 |
|  | *SD* | 22.8 | 22.2 | 22.6 | 21.8 | 21.8 | 22.3 | 18.7 | 23.3 | 18.7 |

SAC-T: Tumoral tissue from serrated adenocarcinoma. CC-T: Tumoral tissue from conventional carcinoma. SAC-N: Normal tissue adjacent to SAC. CC-N: Normal tissue adjacent to CC. MSI: High-level microsatellite unstable CRC.

**3.** Quantitative PCR primers for the quantitation of mRNA expression of *DIO3* and *FOXD2* genes using *β-globin* as a house-keeping gene.

Five μl of 1:5 diluted cDNA was added to the qPCR reaction containing 12.5μl 2X QuantiTect SYBR Green PCR Kit (ref:204145, Qiagen) and 300nM of each primer in a total volume of 25μl. qPCR was performed on a 7500F real time PCR system by Applied Biosystems (Foster City, CA, USA) according to the instruction manual and following the standard protocol: 50ºC 2 min, 95ºC 10 min, 40 cycles of 95ºC 15 sec, 60ºC 1 min and a melt curve stage consisting of 95ºC 15 sec, 60ºC min, 95ºC 30 sec and 60ºC 30 sec. Primers were designed using primer3 software.

| Primer name | Primer sequence 5´-3´ | Annealing  temperature | Amplicon size (bp) |
| --- | --- | --- | --- |
| DIO3-RTF | GCACTTGGTTGGAACGCTAT | 60 | 93 |
| DIO3-RTR | GCCCACCAAGTTCAGTCAAT |
| FOXD2-RTF | CGAGATCTGCGAGTTCATCA | 60 | 108 |
| FOXD2-RTR | TTGACGAAGCAGTCGTTGAG |
| B-ACTIN-RTF | GAGCTACGAGCTGCCTGACG | 60 | 120 |
| B-ACTIN-RTR | GTAGTTTCGTGGATGCCACAG |

**4.** Immunohistochemistry

Details on equipment, antibody purveyor, code, antigen retrieval conditions (buffer, pH, temperature, time), antibody dilution and incubation are as follows: DIO3 gene product (D3): Bechmark Ultra Roche, Thermo scientific (polyclonal), PA5-22886, CC2, acid, 95ºC, 35 min, 1:100, 44 min; FOXD2: Bechmark Ultra Roche, Antibodies-online, ABIN225977, CC1, basic, 95ºC, 60 min, 1:10, 60 min. Endogenous peroxidase activity was blocked using 0.5% H2O2 for 5 min. For visualisation of the antigen, the sections were immersed in 3,3'-diaminobenzidine (DAB) and counterstained with Harris´ haematoxylin for 5 min. Human placenta was used as positive control for D3 and FOXD2 staining [2].

**5.** Statistical analysis

The normality assumption of ANOVAs can be considered to be met, since the sample sizes are sufficiently large and the variables are not strongly skewed [3]. When the assumptions of homoscedasticity, sphericity and matrices equivalence were not supported by the data, the Greenhouse and Geisser-corrected F was used. For polytomous and continuous variables, one-way ANOVAs and bivariate Pearson’s correlation were used, respectively. A multiple linear regression model was performed for predicting the methylation percentage based on significant variables obtained from the bivariate analysis.

**References:**

1. Li LC, Dahiya R. MethPrimer: designing primers for methylation PCRs. Bioinformatics 2002; 18: 1427-1431.

2. Kirk RE. Experimental design: procedures for the behavioral sciences. SAGE: Thousand Oaks, USA, 2013.

3. Tan WY. Sampling distributions and robustness of t-ratio, F-ratio and variance-ratio in 2 samples and ANOVA models with respect to departure from normality. Communications in Statistics - Part A. Theory And Methods 1982; 11: 2485-2511.
