# Supplementary material for: Methylome profiling reveals functions and genes which are differentially methylated in serrated compared to conventional colorectal carcinoma
Source: Clin Epigenetics. 2015 Sep 17;7(1):101. doi: 10.1186/s13148-015-0128-7 (PMC4574063; doi:10.1186/s13148-015-0128-7)
Supplement: Additional file 5: — Multivariate analysis of the clinicopathological and molecular factors associated with FOXD2 methylation (A); DIO3 and FOXD2 mRNA expression in hMSI-H tumoural and normal specimens (B) and external validation using TCGA database showing that MSI-H expressed less FOXD2 than MSI-L/MSS colon carcinomas (C). [file 13148_2015_128_MOESM5_ESM.docx]

**Supplemental material S5.**

**A.** Table showing the coefficients of the multiple regression model

| *n* = 117 | *B coefficients* | *SE* | *B low 95%CI* | *B up 95%CI* | *$\beta$std. coefficients* | *t* | *p* |
| --- | --- | --- | --- | --- | --- | --- | --- |
| Intercept | 32.8 | 3.5 | 25.9 | 39.6 |  | 9.48 | <0.001 |
| Tumoural status | 17.1 | 4.1 | 9.0 | 25.3 | 0.355 | 4.16 | <0.001 |
| MSI | 13.7 | 5.2 | 3.3 | 24.1 | 0.224 | 2.62 | 0.010 |

**B.** Table showing qPCR results for *DIO3* and *FOXD2* expression in hMSI-H tumoral and normal specimens.

| **hMSI-H cases** | | **Tumoral** | **Non-tumoral** |
| --- | --- | --- | --- |
| n | | 8 | 5 |
|  | *Median*±*SD* | 0.002±0.008 | 0.036±0.017 |
| *DIO3* | Mann-Whit. *U* |  | 2 |
|  | *p* |  | 0.008 |
|  | *Median*±*SD* | 0.0007±0.003 | 0.0079±0.052 |
| *FOXD2* | Mann-Whit. *U* |  | 4 |
|  | *p* |  | 0.019 |

**C.** External validation. *FOXD2* expression levels according to MSI status retrieved from the TCGA database colon carcinoma cases.

| ***FOXD2* expression** | | **N** | **Mean±SD** | **p value** |
| --- | --- | --- | --- | --- |
| MSS/MSI-L | | 263 | 7.759±1.144 |  |
| MSI-H | | 61 | 7.065±1.274 | <0.001 |
|  |  |  |  |  |
