# Supplementary material for: Methylome profiling reveals functions and genes which are differentially methylated in serrated compared to conventional colorectal carcinoma
Source: Clin Epigenetics. 2015 Sep 17;7(1):101. doi: 10.1186/s13148-015-0128-7 (PMC4574063; doi:10.1186/s13148-015-0128-7)
Supplement: Additional file 7: — Distribution of the CpG sites studied in the FOXD2 gene and correlation table of the methylation percentages at the 3´ UTR CpG sites with FOXD2 mRNA expression. [file 13148_2015_128_MOESM7_ESM.doc]

**Supplemental material S7.** *FOXD2* CpGs metylation

**UTR and coding region of *FOXD2* showing the regions and CpG sites of study**

Legend:

- Untranslated Region
- Region 1 of analysis (5´UTR)
- Region 2 of analysis (5´UTR)
- Translated sequence
- Region 3 of analysis (3´UTR)
- CpGs pyrosequenced
- **CpGs included as probes in the methylome array**

CTTTCCTAAATGGCCTAGAATATGGCTACACTTATACATCAGTTCTGAGGTAGGTGTTACTAGCCTCATTTTTACAAAGGCAGAAACAAAG**CG**CAAAGAACCTTGCCCAAGGTGACAGATAGGGTAAGTAGTGAAGGCAGGATTGGAACCCCAGGAGCCTGACTTAAGGTCGCTGCTCTTAACTACTATGCAGACCAGGAAACTGAGGCTCCGAAGCGGGGAAGTCACCTAATGAGGGTCACACAGCCTTGCTACAGCGGAGGCAGCTGGGATTC**CG**GCCT**CG**CACGCCCGAGCTTTAGCCACTGTGCGGCGCTGT**CG**GGGCAGCAGCCCAGGCATCCGCTCAGTTCCTGCTCACGTCCAGCGCCCATCTCCCCTTC**CG**TGCCCCAGAGGCTGAAATGGGCCCAATCTGTGTTGAAGCTGCTTGTTTCTGGGTCCTGGAGGGAGGCCGGGGGAGAAAGAGGTCTAATAGGATGGGCAGGAACCCGGCTAGCGAGAAACAAGGCACAGGGCCAACCTTAGTTGCACAAGACTTTTGAGAGATGAAGGCAACACTTGGTGGAGGTCAGGGTTCACTGAGGGGGTGTCGGGCAGGGGGCGGAGGAGGTCCTGCCGCCTGTCGGTGGGGCCCAGAACCCTGGGAGCCAGGAAGGTGGGGGGTGTCCAGTCCTCAGGGCAACTGGGGCCAGGGAGAGGGGAGGGAGAAATTAAGCGGATTCCCAGACCCATGAACAGACATCCAGACAAATGCCGTTTCCACAGAGACTCACGGAGAGTGAGATAGTCACAGAGACTGAGACAAACGGATGCCGAGATCCAGAGGGTCGGAGAACAGGCAAACT**CG**GAGCAGTAGAC**CG**GCCCGCGCCCCGGGAACGCGGCCACAGCGGCCGGCGCTCTGCCCGGCCTCTCCGCGCGGAGAGGGGTGGCGGGCCCGACAGCGGAGGGAGGAGAGGGCGCCGCCGCAATCTCACCTCTCCCAGGCCCAAGGCTGCGGGCGCCAAGTTCGCTCCTGGTGATCACTCGAATGCGTCTCCCCTCGGGCTGGAAGCCGTGCGGGCTCGGGTGGGCGCCGGCGGGCCGCGCTGGGGCGGCGTGGGTCCCGGCGTCTCAGCCTCGCGCTCCCACTGCGCTTCGGCCCGGTGGCCCGGGCGCCGCCTTGGGGAGAGGACGGGTGGGGACCGCCGCCACTTCCCTGGCTGCGGCTGGCGCCCGAGTGAGCCTTAACATCCAGGGGCTGAGCGTTCTGAAGGCGGCGGCTTCAGGGAGCACAGGGTGCAGGAGCGGCGGCGAAGACAAGGGCCCGCCTCCGGCCACTCGAGCCCAGCTCCCGCCGCGGCGGCGGTTTGTTCCCGCCGGGTCCCTCAGCGGAGGCGCTACGCC**CG**CCCCTGT**CG**CCT**CG**CCCCACCCCGCCCAGGGAGCTCCGCCCTAGCCCGCAGCTCTTCCGCCTTAGGCAGCCGCTAGG**CG**GGAGGGACAATCCCCCACCACCAACCACTGCCACCCCGAGGGGACTAGGGGCTGAGGCCCGCCCAGGTAAGGGAAAGCCTCAGCTCCTTCCGTTGCGCCCCAGCGGCGGGTCCCAGCTCGGATTCCCGGGGTAGTGGCGGGGGCCGCCGGCGGGTCGTGCCCTGGAAGGTGAGCGCGGCCGAGCTGGGCCGCCAGGGGGCGCTGCGGAGCCGGGGGACACCCCTCCCTGCCTGCCTCAGTCCCCCGCCCCCTCCCCGCCCGCGCGCAAAACGCACTCGCCCCAGAGGCAGCGCGGCCGAGCCCGAGCCGCTGCCGGAGCGGAGCCGGAGAGTGGCGGCGGCGGCGGCAG**CG**GCACCATGACCCTGGGCAGCTGCTGCTGCGAGATCATGTCCTCCGAGAGCTCCCCGGCCGCGCTGTCCGAGGCCGACGCAGACATAGACGTGGTGGGCGGCGGCAGCGGCGGGGGGGAGCTCCCAGCTCGCTCCGGGCCCCGCGCCCCCCGGGACGTGCTCCCCCACGGCCACGAGCCTCCCGCGGAGGAAGCCGAGGCAGACTTAGCCGAGGACGAGGAGGAGTCTGGTGGCTGCTCGGACGGCGAGCCCCGCGCTCTGGCGTCCCGGGGGGCGGCGGCCGCAGCGGGGAGCCCGGGGCCAGGCGCCGCGGCGGCCCGCGGCGCAGCGGGGCCCGGGCCGGGACCGCCGTCGGGGGGCGCGGCGACG**CG**GAGCCCGCTGGTGAAGCCGCCCTACTCGTACATCGCGCTCATCACCATGGCCATCCTGCAGAGCCCCAAGAAGCGGCTGACGTTGAGCGAGATCTGCGAGTTCATCAGCGGCCGCTTCCCCTACTACCGGGAGAAGTTCCCCGCCTGGCAGAACAGCATCCGCCACAACCTCTCTCTCAACGACTGCTTCGTCAAGATCCCCCGCGAGCCGGGCAACCCGGGCAAGGGCAACTACTGGACGCTGGACCCGGAGTCGGCCGACATGTTCGACAACGGCAGCTTCCTGCGGCGT**CG**CAAGCGCTTCAAGCGGCAGCCCCTGCCGCCGCCGCACCCACACCCGCACCCTCACCCGGAGCTGCTGCTGCGTGGCGGGGCCGCGGCGGCGGGGGATCCCGGCGCTTTCCTGCCCGGCTTCGCTGCCTACGGCGCCTACGGCTACGGCTACGGGCTGGCTCTCCCGGCCTACGGCGCACCCCCGCCGGGGCCGGCCCCGCATCCGCACCCGCACCCGCACGCCTTCGCTTTCGCCGCGGCAGCCGCCGCCGCTCCTTGCCAGCTGTCGGTACCCCCAGGCCGCGCCGCCGCGCCTCCACCCGGACCTCCGACGGCCTCGGTGTTCGCAGGCGCGGGATCGGCCCCAGCTCCTGCGCCTGCCTCAGGCTCGGGCCCGGGCCCGGGCCCCGCAGGCCTGCCCGCCTTCCTGGGCGCGGAGCTGGGCTGCGCCAAAGCCTTCTACGCGGCGTCCCTGAGTCCTCCCGCAGCCGGCACCGCGGCGGGTCTGCCCACCGCACTTCTGCGCCAGGGCCTCAAGACGGACGCGGGCGGTGGTGCAGGCGGCGGGGGCGCCGGGGCAGGGCAGAGGCCTTCCTTCTCTATAGACCACATCATGGGCCA**CG**GTGGCGGCGGGGCAGCACCCCCGGGCGCCGGCGAGGGCTCTCCGGGACCGCCATTCGCGGCAGCCGCGGGTCCTGGGGGCCAAGCCCAGGTCTTGGCCATGCTGACTGCTCCGGCCCTGGCTCCCGTTGCTGGCCACATTCGCCTCTCGCATCCCGGGGACGCGCTGCTGTCCTCAGGGTCCCGGTTTGCCAGCAAAGTCGCCGGCCTTAGTGGCTGCCACTTCTGACCGCAGCAGGCCCAGGGCCGGTTAGGTCCGCACTCCTCAGCCTCTCCCGGGAGTTCCTGCGGTCCCAGCGGAACTCAGGGAGTCTATTTATGAAGTCTCCAGACCTTGGGCCGGCACGCGTGACACGGCACTTCAGGCTCCA**CG**CACAGAATCTCGCAGATAGTTGGGACTAAGCGGGCTCTATCGCTCAGGGCGACAGGCCCGGGGCTACGCGAAGAAGT**CG**CAGGCCAAGATTCTTTACAGTTTGAGAAATAAAAGCAGGGGGGTGGGGGCTTCGTTTTTTTCCCTGCCTCTGCGCCTCTCGGGGAACACATTCCGGGAGAGATGCCTGGCCAGGCTCCACGGATCCCGCCAGAAACACCAACAGAGGGTCTCCCTTTCTGCCTTTCCCCTCTCACTTCTTCCCCAACGT

**Correlation between the methylation percentage of *FOXD2* 3´UTR CpG sites and *FOXD2* mRNA expression in 26 cases (except otherwise specified)**

| **Meth-mRNA correlations** | | **Spearman's Correlation** | |  |
| --- | --- | --- | --- | --- |
| CpG1 site | *r* | -0.467* | |  |
|  | *p* | 0.016 | |  |
| CpG2 site | *r* | -0.518** | |  |
|  | *p* | 0.007 | |  |
| CpG3 site | *r* | -0.482* | |  |
|  | *p* | 0.013 | |  |
| CpG4 site | *r* | -0.488* | |  |
|  | *p* | 0.011 | |  |
| CpG5 site | *r* | -0.480* | |  |
|  | *p* | 0.013 | |  |
| CpG6 site | *r* | -0.443* | |  |
|  | *p* | 0.024 | |  |
| CpG7 site | *r* | -0.486* | |  |
|  | *p* | 0.012 | |  |
| CpG8 site | *r* | -0.404* | |  |
| (n=25) | *p* | 0.045 | |  |
| CpG9 site | *r* | -0.352 | |  |
| (n=25) | *p* | 0.084 | |  |
| * p< 0.05 (two-sided) | | |  | |
| ** p< 0.01 (two-sided) | | |  | |

Due to the non-normal distribution of qPCR results Spearman’s correlation was chosen instead of Pearson´s.
